# Metastatic Disease Mimicking Osteomyelitis in the Foot

**DOI:** 10.7759/cureus.61608

**Published:** 2024-06-03

**Authors:** Linda Youn, Amber Kuta, Karina S Shendrik

**Affiliations:** 1 Department of Internal Medicine, St. Bernards Medical Center, Jonesboro, USA

**Keywords:** cisplatin, pembrolizumab, calcaneal bone, cervical cancer, metastatic disease, acrometastasis, osteomyelitis

## Abstract

Cervical cancer most commonly spreads hematogenously to the lungs, liver, and bone. However, it rarely metastasizes to the foot. There is only one other case of cervical cancer with metastasis to the foot. In addition, the initial imaging of metastatic disease has difficulty in differentiating from infectious or other inflammatory processes, particularly in a clinical setting highly suspicious of infectious sources. Here, we present a rare case of cervical cancer metastasizing to the calcaneus masquerading as osteomyelitis, highlighting the importance of diagnostic imaging in conjunction with histological confirmation.

## Introduction

The most common sites for the hematogenous spread of cervical cancer are the lungs, liver, and bone. However, bone metastasis to the foot is rare [1-3]. The incidence of cervical cancer metastasizing to the bone has been reported to range from 1.8% to 6.6% [4,5]. The most common sites involve the proximal skeleton with the thoracic spine, lumbar spine, and ribs making up the majority [5]. Patients can present with symptoms resembling an inflammatory or infectious etiology including arthritis, gout, and osteomyelitis.

The lesion may not present with clear radiographic signs in the early stages of disease [6]. However, the penumbra sign, a higher signal intensity feature of the granulation tissue lining the abscess cavity on T1-weighted magnetic resonance (MR) images, has been shown to have 73.3% sensitivity and 99.1 specificity for osteomyelitis, which could support excluding the presence of tumor [6].

Here, we describe a case of cervical cancer metastasis to the calcaneus mimicking osteomyelitis.

## Case presentation

**Figure 1:**
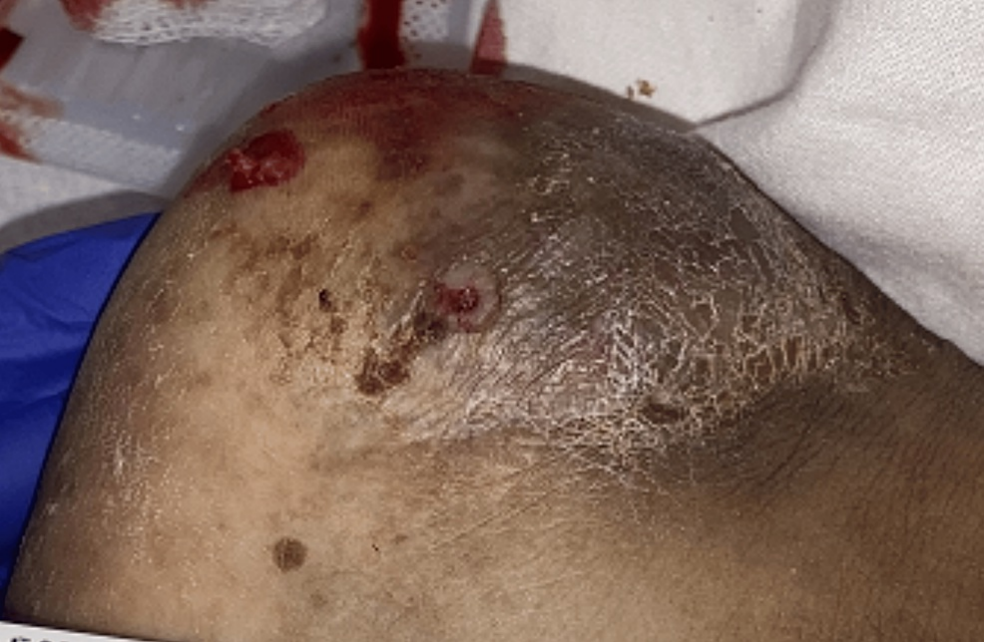
Photo of distal left ankle with fluctuant mass measuring 0.2 × 0.2 × 2.1 cm.

A female in her 40s presented with generalized pain, weakness, and tachycardia. The patient was known to have cervical cancer with known metastasis to the lymph nodes, left adrenal gland, and bladder. She also had urinary obstruction secondary to metastatic disease causing bilateral hydronephrosis requiring bilateral nephrostomy tubes. The patient was on a treatment regimen of pembrolizumab and cisplatin. She had recently been admitted for bacteremia and sepsis secondary to a staphylococcal urinary tract infection. The patient was found to be septic again with the source of infection being suspected to be either a urinary or a foot abscess (Figure 1).

**Figure 2:**
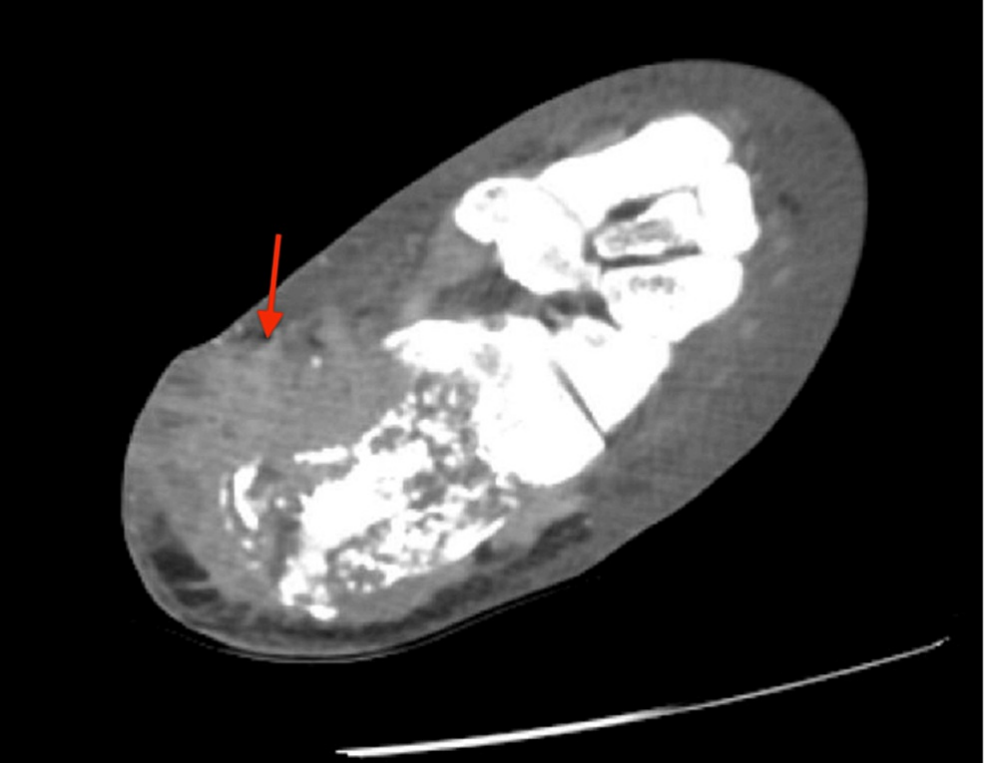
CT of the left leg with contrast showing diffuse subcutaneous fat stranding. Fluid collection within the distal Achilles tendon measuring 1.5 × 1.5 × 2.4 cm. Peripheral fluid collection of the posterior medial heel region within the subcutaneous fat measuring 4.0 × 1.7 × 2.3 cm (red arrow). CT: computed tomography

She was started on broad-spectrum antibiotics. A computed tomography (CT) of the left leg was performed showing an extensive comminuted fracture of the calcaneus with incomplete healing suggestive of osteolysis and bone loss, multiloculated fluid collection within the subcutaneous fat posterior medial to the calcaneus concerning for an abscess, and additional fluid collection within the distal Achilles tendon (Figure 2). Foot fluid collection cultures were negative, and subsequent foot surgical cultures were negative. However, these cultures were obtained more than 48 hours after the initiation of antibiotics. The patient ultimately underwent surgical debridement. Surgical pathology results showed immunohistochemical staining positive for p40 and cytokeratin 7 (CK7) and morphologic features consistent with dermal primary with squamous differentiation strongly suggestive of metastatic disease.

**Figure 3:**
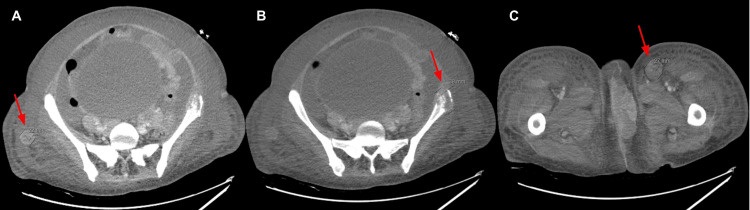
CT of the abdomen/pelvis showing multiple soft tissue masses in the right gluteal fat with the largest measuring 2.2 cm in axial view indicated by the red arrow (A), expansive lytic lesion in the left iliac measuring at least 3.9 cm in axial view indicated by the red arrow (B), and bulky left inguinal adenopathy with one node measuring at least 2.7 cm in axial view indicated by the red arrow (C). CT: computed tomography

CT of the abdomen and pelvis was obtained because of persistent leukocytosis and fevers despite antibiotic treatment, which showed large cervical mass extending into the left vaginal folds with innumerable pulmonary and hepatic metastatic lesions, additional metastatic disease involving left inguinal nodes, soft tissue nodules in the right gluteal fat, lytic left iliac lesion, and ascites with diffuse subcutaneous fat stranding suggesting third spacing (Figure 3), bilateral pleural effusions.

Although there was no progression of disease from previous imaging one month prior, due to continued large tumor burden, poor performance status, other comorbidities, and acute illness, the patient was no longer deemed a candidate for aggressive treatment. Hospice care was recommended, and soon after, the patient opted to pursue comfort measures. The patient ultimately expired.

## Discussion

Acrometastasis is the term used to describe metastasis to the hands and feet. Acrometastasis is relatively rare with studies showing an incidence of merely 0.007%-0.3%. Furthermore, less than half of those cases involve the feet. Signs and symptoms typically found with acrometastasis, such as soft tissue swelling and pain, are nonspecific and can easily be confused for other medical conditions such as gout, rheumatoid arthritis, ligamentous sprains, osteoarthritis, Paget’s disease, or infections such as osteomyelitis. Skeletal surveys and whole-body CT commonly excluded distal extremities, making the identification of metastasis to these areas difficult to detect. The most commonly associated primary cancers are lung, gastrointestinal, and genitourinary tumors [7]. Our patient had acrometastasis from a cervical primary.

Cervical cancer is the second leading cause of cancer mortality in females between the ages of 20 and 39 years old. Patients with cervical cancer have a poor prognosis with a mean survival of 8-13 months in those with metastatic disease. The presence of multi-site metastasis worsens poor prognosis. Metastatic disease in cervical cancer occurs through hematogenous and lymphatic routes in the majority of cases associated with cancer-related death. The most common single site of metastasis is the lung as it is believed to have a microenvironment that is favorable for cervical cancer cells [8]. The next most common site is the liver, followed by the bone for single-site metastasis. Bone metastasis has an incidence of 2.5% with the most frequent site being the vertebral column, particularly in the lumbar region. Bone metastasis is associated with poor prognosis, and prompt identification is paramount in preventing pathologic fractures and disability.

From our literary review, we found one other case of cervical cancer with metastasis to the calcaneal bone. In that case, the patient underwent both plain film imaging, which noted an osteolytic lesion, and magnetic resonance imaging (MRI), which noted a 4 cm mass located on the calcaneus. Their patient also underwent 2-deoxy-2-[18F]fluoro-D-glucose positron emission tomography/computed tomography (18F-FDG PET/CT), which showed an increased uptake in the calcaneus. Ultimately, a biopsy was done on the mass, which revealed squamous cell carcinoma [9].

Skeletal scintigraphy or 18F-FDG PET/CT is commonly used to identify bone metastases. X-rays, CT scans, and MRI can also be used to evaluate lesions found on skeletal surveys or FDG PET/CT to help differentiate inflammation or osteoporotic lesions from metastasis [9-11]. Osteomyelitis may also appear different in imaging based on chronicity. The preferred modalities for initial evaluation imaging are plain radiography as the first line and MRI as the second line. Radiography, although first line, has generally poor sensitivity especially in the acute phase. MRI has better sensitivity ranging from 60% to 100% with lower sensitivities noted in studies done without contrast [12].

However, studies have shown that various imaging modalities are limited in differentiating acute reactions caused by inflammation or infection and malignancy. Pruckmayer et al. report a case of bone metastasis with superimposed osteomyelitis in which the patient underwent orthopantomography, whole-body bone scintigraphy, antigranulocyte immunoscintigraphy, single-photon emission computed tomography (SPECT), and CT scan with results suggestive of osteomyelitis. Their patient was placed on antibiotics, and it was decided to proceed with a biopsy to rule out bone metastasis. The biopsy showed metastatic prostate cancer with massive leukocytic invasion. They acknowledge in this case that imaging modalities were ineffective in identifying the underlying metastasis and that ultimately invasive diagnosis was mandatory to differentiate osteomyelitis from superinfected bone metastasis [13].

As seen in our case and the cases above, biopsy and histopathology were required as clinical presentation and imaging were unspecific for definitive diagnosis. However, a meta-analysis of 18-FDG PET/CT, 18F-FDG PET, MRI, and bone scintigraphy by Qu et al. found that 18F-FDG PET/CT has a higher diagnostic value for diagnosing bone metastasis from lung cancer than any other imaging modalities [11]. A similar meta-analysis of 18-FDG PET/CT, 18F-FDG PET-MRI, and bone scintigraphy regarding bone metastasis in patients with cervical cancer may further prevent the need for invasive procedures for definitive diagnosis.

## Conclusions

This unique case of cervical cancer metastasizing to the calcaneus highlights the importance of diagnostic imaging in conjunction with histological confirmation. Particularly in the setting of late-stage cancer, timely diagnosis and appropriate management would provide symptomatic relief, as well as potentially curb serious morbidity.
